# Effect of 1-Kestose on Lipid Metabolism in a High-Fat-Diet Rat Model

**DOI:** 10.3390/nu17081362

**Published:** 2025-04-16

**Authors:** Kento Kuramitsu, Mikoto Kubo, Felicia Cindy, Takahiro Shibata, Yoshihiro Kadota, Yasuyuki Kitaura

**Affiliations:** 1 Department of Applied Biosciences, Graduate School of Bioagricultural Sciences, Nagoya University, Nagoya 464-8601, Aichi, Japan; kuramitsu.kento.m3@s.mail.nagoya-u.ac.jp (K.K.);; 2 B Food Science Co., Ltd., Chita 478-0046, Aichi, Japan; 3 Department of Applied Biosciences, Chubu University, Kasugai 487-8501, Aichi, Japan

**Keywords:** 1-kestose, cholesterol, bile acids, lipid metabolism, gut microbiota

## Abstract

**Objectives:** Hyperlipidemia is a risk factor for various diseases. Identifying food components that can help reduce the levels of blood lipids, such as cholesterol and triglycerides, is a global research priority. It has been reported that 1-Kestose, a fructooligosaccharide, can reduce blood cholesterol and triglyceride levels in rats; however, the underlying mechanisms remain unclear. Therefore, we aimed to elucidate the effects of 1-kestose supplementation on lipid metabolism and the gut environment in rats. **Methods**: Twenty male Sprague–Dawley rats (age 8 weeks) were provided 1-kestose-containing water and were maintained for two weeks. After dissection, the blood components, hepatic gene expression, gut microbiota, and bile acid composition in the cecal contents of the rats were analyzed. **Results**: The 1-Kestose intake reduced plasma cholesterol and triglyceride levels. Additionally, an increase in cytochrome P450 family 7 subfamily A member 1 mRNA expression, a key gene for bile acid synthesis in the liver, and a decrease in lipid synthesis-related mRNA expression were observed. In the cecum, the levels of deconjugated bile acids, which are involved in the regulation of lipid synthesis, were increased. Furthermore, the 1-kestose intake altered the gut microbiota in the cecum, leading to an increase in the abundance of specific bacteria, such as *Bifidobacterium*, which are involved in the deconjugation of conjugated bile acids. **Conclusions**: The intake of 1-kestose alters the gut microbiota and bile acid metabolism in the cecum, potentially influencing lipid metabolism in the host.

## 1. Introduction

Cardiovascular disease is a major health issue and is the leading cause of death worldwide [1,2]. Hyperlipidemia, characterized by high blood cholesterol and triglyceride levels, is a significant risk factor for cardiovascular disease as it impairs blood and oxygen transport [3,4,5]. Cholesterol-lowering medications, which are typically prescribed to treat hyperlipidemia, include statins, which inhibit cholesterol synthesis, and ezetimibe, which blocks cholesterol absorption in the intestine. However, these drugs may cause side effects such as muscle pain, weakness, and liver inflammation [6,7,8]. Thus, the development of new drugs and the identification of food ingredients that lower cholesterol and triglycerides are urgently required.

In the liver, cholesterol is converted into primary bile acids via a series of reactions catalyzed by the rate-limiting enzyme cytochrome P450 family 7 subfamily A member 1 (CYP7A1). These bile acids are then conjugated with glycine or taurine, secreted into the bile, and released into the intestinal tract, where they facilitate lipid digestion and absorption by the intestinal epithelial cells. Although most bile acids are reabsorbed before passing through the ileum, certain intestinal bacteria can deconjugate them, thereby modifying the enterohepatic circulation of bile acids. Triglycerides are synthesized in the liver and adipose tissues from acetyl-CoA, a process mediated by lipid biosynthesis-related genes. Sterol regulatory element-binding protein-1 (SREBP1) serves as a multifunctional transcription factor for various lipid biosynthesis-related genes [9].

The gut microbiota play a crucial role in maintaining host health not only by fermenting dietary components but also through various other functions, such as modulating the immune system, producing vitamins, and regulating metabolism [10]. Studies using germ-free mice and fecal transplantation experiments have revealed that the gut microbiota are involved in regulating blood cholesterol and triglyceride levels [11,12]. Certain intestinal bacteria harbor genes that facilitate bile acid deconjugation and dihydroxylation, which can influence bile acid metabolism in the gut. It has been shown that gut bacteria convert primary bile acids into secondary bile acids, which can activate nuclear receptors in the host and influence metabolism and immune responses. For example, the secondary bile acid lithocholic acid has been reported to exert anti-inflammatory effects through the vitamin D receptor, while it is also known to be a risk factor for colorectal cancer [13,14,15]. Bile acids, via the activation of farnesoid X receptors (Fxr) in the liver, regulate *Cyp7Aa* and *Srebp1* expression [16,17,18]. Therefore, modulating the gut microbiota represents a potential strategy for lowering blood cholesterol and triglyceride levels [19].

Prebiotics are defined as “a substrate that is selectively utilized by host microorganisms conferring a health benefit” [20]. Commercially available fructooligosaccharide consists of different ratios of 1-kestose, nystose, and fructofuranosylnystose, which contain 1–3 fructose monomers linked to sucrose via β-2,1 glycosidic bonds [21]. Animal studies in mice and rats have reported that 1-kestose improves insulin resistance, reduces adipose tissue inflammation, and alleviates allergies [22,23,24,25]. Clinically, 1-kestose supplementation has improved conditions such as tolerance-associated colitis and food allergies [26,27]. Additionally, studies on rats have reported that 1-kestose intake tends to reduce blood cholesterol and triglyceride levels [28]. However, the mechanism by which 1-kestose reduces lipid levels remains unclear. Therefore, we investigated the effects of 1-kestose intake on the gut microbiota and host lipid metabolism in rats fed a high-fat diet. We found that 1-kestose intake alters the gut microbiota and bile acid metabolism in the cecum, thereby modulating lipid metabolism in the host.

## 2. Materials and Methods

### 2.1. Animal Experiments

All animal experiments were approved by the Animal Care Committee of the Nagoya University Graduate School of Bioagricultural Sciences (approval number: A230037). Twenty male Sprague–Dawley rats aged 7 weeks (body weight, 210–230 g) were obtained from Japan SLC (Hamamatsu, Japan) and individually housed in wire-mesh cages in a conventional animal room at a controlled temperature (23 ± 2 °C) with a 12 h light–dark cycle (light exposure beginning at 8:00 A.M.). After acclimatization to the animal room over 1 week, the rats were fed a high-fat diet (D12492, Research Diets, New Brunswick, NJ, USA) that provided 60% energy from fat, 20% energy from protein, and 20% energy from carbohydrates. The rats were then assigned to two groups treated with (KES [+]) or without (KES [−]) 1-kestose (each, n = 10); tap water with and without 4% (*w*/*v*) 1-kestose (purification, >95%; B Food Science, Chita, Japan) was administered to the KES (+) and KES (−) groups, respectively. The rats were provided with free access to the corresponding experimental diets and water for 2 weeks. On the final day of the experiment, eight rats were fasted for 8 h, euthanized via exsanguination under isoflurane anesthesia, and blood samples were collected into heparinized tubes to analyze post-heparin plasma. The liver and cecum contents were removed and frozen in liquid nitrogen; the pH of the cecum contents was measured, as previously described [28]. All obtained tissues were stored at −80 °C until use.

### 2.2. Measurement of Blood Components

Glucose, cholesterol, triglyceride, and free fatty acid levels in the plasma of fasted rats were measured using CII test WAKO kits (Fujifilm, Osaka, Japan), whereas insulin levels were determined using the Rat Insulin ELISA Kit (Morinaga, Kanagawa, Japan).

### 2.3. Analysis of mRNA Gene Expression in Liver Tissues by Quantitative Reverse Transcription Polymerase Chain Reaction

Approximately 30 mg of liver tissue was powdered in liquid nitrogen and used to prepare total RNA. Total RNA was extracted and purified from liver tissues using ISOGEN II (Nippon gene, Tokyo, Japan). Purified total RNA was reverse-transcribed to cDNA using the ReverTra Ace qPCR RT kit (Toyobo, Osaka, Japan). DNA amplification was performed in a final 20 µL reaction mixture containing 15 ng of cDNA, optimized specific primers [29,30,31] (Table 1), and the THUNDERBIRD SYBR qPCR mix (Toyobo) using StepOnePlus (Thermo Fisher Scientific, Waltham, MA, USA) according to the manufacturer’s instructions. The polymerase chain reaction (PCR) protocol included an initial denaturation at 95 °C for 1 min, followed by 40 cycles of amplification (95 °C for 10 s, 60 °C for 1 min, 72 °C for 1 min), and a final melting curve analysis. Results are expressed as fold increase relative to the −KES after normalization to the glyceraldehyde-3-phosphate dehydrogenase gene expression level.

### 2.4. Measurement of Metabolite Levels in the Cecum

The concentration of each bile acid in the cecum was measured using ultra-performance liquid chromatography–mass spectrometry (MS). Approximately 20 mg of the weighed cecal contents was combined with 20 µL of 100 µg/mL 5β-cholanic acid as an internal standard. After adding 500 µL of 99.5% methanol as an extraction solvent, the mixture was vortexed for 15 s. Following centrifugation (16,000× *g*, 3 min, 4 °C), 400 µL of the supernatant was transferred to an Eppendorf tube, filtered, and used for liquid chromatography (LC)/MS analysis with a 2.1 mm × 150 mm C18 column (Thermo Fisher Scientific, Waltham, MA, USA). Results are expressed as the fold increase relative to the −KES after normalization to the 5β-cholanic acid level.

### 2.5. 16S rRNA Sequencing

Frozen cecum samples were thawed on ice, and DNA was extracted from 20 mg of each sample using ISOSPIN Fecal DNA (Nippon gene). The DNA concentration of each sample was estimated using an ND-1000 spectrophotometer (NanoDrop Technologies, Wilmington, DE, USA), and the final concentration of each DNA sample was adjusted to 12.5 ng/µL. PCR amplification of the V3–V4 region of bacterial 16S rRNA genes. Multiplex sequencing was performed as previously described using the following primers: forward primer, 5′-ACACTCTTTCCCTACACGACGCTCTTCCGATCTXXXXXCCTACGGGNGGCWGCAG-3′ and reverse primer, 5′-GTGACTGGAGTTCAGACGTGTGCTCTTCCGATCTXXXXXGACTACHVGGGTATCTAATCC-3′ [24,32]. QIIME2 (version 2023.5) was used for 16S rRNA gene expression analysis. The reads were quality filtered, trimmed, merged, denoised, chimera-filtered, and binned to yield sequence variants using DADA2. Sequence variants were aligned to the Silva reference database 138. Alpha diversity was calculated, and principal coordinate analysis of variance with weighted UniFrac distances was performed using the “qiime diversity core-metrics-phylogenetic” command. Beta diversity was assessed using weighted UniFrac distances and the “qiime diversity beta-group-significance” command. A systematic survey of the community was performed using Phylogenetic Investigation of Communities by Reconstruction of Unobserved States (PICRUSt2) to identify the functional genes of the intestinal bacteria. Gene counts in each sample were normalized by calculating the relative abundance of each gene per total gene count in the inferred metagenome for the sample [33].

### 2.6. Statistical Analysis

Data are presented as means ± standard deviations. Statistical analyses were performed using Prism version 10 (GraphPad Software, San Diego, CA, USA). Unpaired *t*-tests were used to analyze normally and equally distributed data. For non-normally distributed data, the Mann–Whitney U test was used. Beta diversity and associated *p*-values were determined using permutational multivariate analysis of variance. Analysis of the composition of microbiomes (ANCOM) was used to determine differentially abundant taxa [34]. Spearman’s rank correlation coefficient was used to assess the relationships between variables. Differences were considered significant at *p* < 0.05.

## 3. Results

### 3.1. 1-Kestose Suppressed Weight Gain and Reduced Blood Lipid Levels

**Figure 1:**
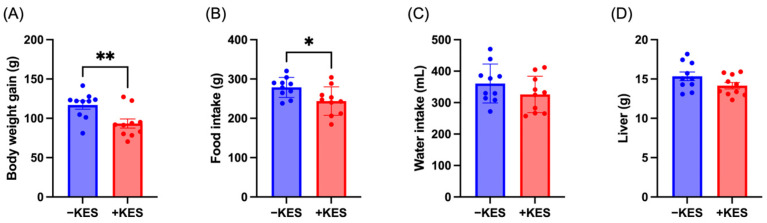
(**A**) Body weight gain after 2 weeks of feeding, (**B**) food intake, and (**C**) water intake along with (**D**) liver weight. Data are presented as means ± standard errors (n = 10 animals per group). * *p* < 0.05, ** *p* < 0.01. +KES, 4% (*w*/*v*) 1-kestose treatment; −KES, control.

High-fat diet intake induces weight gain and an increase in blood lipid levels in rats [35,36]. Regardless of feeding the rats a high-fat diet for two weeks, their body weight and food intake were significantly reduced in the +KES group (Figure 1A,B). There were no significant differences in water intake or liver weight between the two groups (Figure 1C,D).

**Figure 2:**
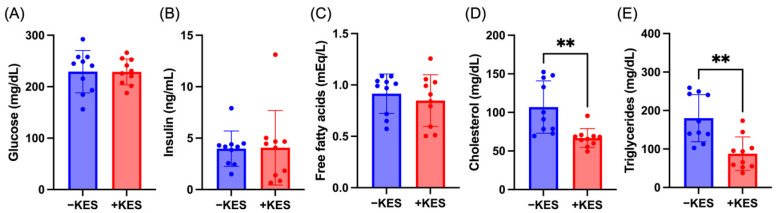
Concentrations of (**A**) glucose, (**B**) insulin, (**C**) free fatty acids, (**D**) cholesterol, and (**E**) triglycerides in plasma. Data are presented as means ± standard deviations (n = 10 animals per group). ** *p* < 0.01. +KES, 4% (*w*/*v*) 1-kestose treatment; −KES, control.

There were no significant changes in the plasma glucose, insulin, and free fatty acid levels after 8 h of fasting (Figure 2A–C). However, the plasma cholesterol and triglyceride levels were significantly reduced in the +KES group (Figure 2D,E).

### 3.2. 1-Kestose Altered mRNA Gene Expression in Liver Tissues

**Figure 3:**
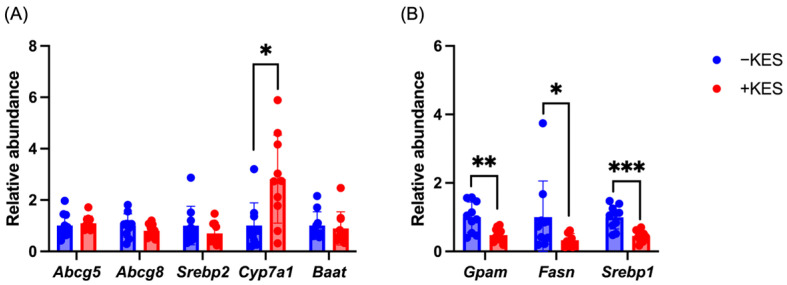
(**A**) The relative abundance of cholesterol metabolism-related genes, including *Abcg5*, *Abcg8*, *Srebp2*, *Cyp7a1* and *Baat*, in liver tissues of rats. (**B**) The relative abundance of triglyceride metabolism-related genes, including *Gpam*, *Fasn* and *Srebp1*, in liver tissues of rats. Data are presented as mean ± standard deviation (n = 10 animals per group). * *p* < 0.05, ** *p* < 0.01, *** *p* < 0.001. *Abcg5*, ATP-binding cassette sub-family G member 5; *Abcg8*, ATP-binding cassette sub-family G member 8; *Srebp2*, sterol regulatory element-binding protein 2; *Cyp7a1*, cytochrome P450 family 7 subfamily A member 1; *Baat*, bile acid-CoA: amino acid N-acyltransferase; *Gpam*, glycerol-3-phosphate acyltransferase mitochondrial; *Fasn*, fatty acid synthase; *Srebp1*, sterol regulatory element-binding protein 1; +KES, 4% (*w*/*v*) 1-kestose treatment; −KES, control.

The mRNA expression of cholesterol metabolism-related genes in the liver was analyzed using quantitative reverse transcription PCR. There were no significant differences in the mRNA expression of ATP-binding cassette subfamily G members 5 (Abcg5) and 8 (Abcg8), Srebp2, and bile acid-CoA:amino acid N-acyltransferase (Baat) between the two groups (Figure 3A). However, Cyp7a1 expression was significantly increased in the +KES group.

We examined the mRNA expression of triglyceride metabolism-related genes in the liver. The expression of genes involved in lipid synthesis, such as of glycerol-3-phosphate acyltransferase mitochondrial (Gpam) and fatty acid synthase (Fasn), were significantly reduced in the +KES group (Figure 3B). Additionally, the expression of Srebp1, a regulatory transcription factor for lipid synthesis genes, significantly decreased in the +KES group.

### 3.3. 1-Kestose Intake Altered the Bile Acid Composition in Cecal Contents

**Figure 4:**
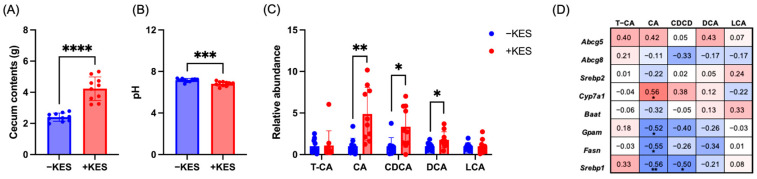
Cecal content (**A**), weight, and (**B**) pH. (**C**) Concentrations of T-CA, CA, CDCA, DCA, and LCA in cecal contents. (**D**) Correlation analysis between bile acids and mRNA expression levels was performed using Spearman’s correlation. Data are presented as the mean ± standard deviation (n = 10 animals per group). * *p* < 0.05, ** *p* < 0.01, *** *p* < 0.001, **** *p* < 0.0001 +KES, 4% (*w*/*v*) 1-kestose treatment; −KES, control; T-CA, taurocholic acid; CA, cholic acid; CDCA, chenodeoxycholic acid; DCA, deoxycholic acid; LCA, lithocholic acid.

In the +KES group, the weight of the cecal content significantly increased, whereas the pH of the cecal content significantly decreased (Figure 4A,B). We evaluated the bile acid composition in the cecal content using LC-MS. Levels of the conjugated bile acid taurocholic acid did not change significantly (Figure 4C). Additionally, levels of the primary bile acids—cholic acid (CA) and chenodeoxycholic acid (CDCA)—and the secondary bile acid—deoxycholic acid (DCA)—significantly increased in the +KES group. In contrast, no significant changes were observed in levels of lithocholic acid, a secondary bile acid. Spearman’s correlation analysis was used to evaluate the associations between bile acids and the mRNA expression levels. As a result, positive correlations were observed between CA and *Cyp7a1*. In contrast, negative correlations were found between CA and *Gpam*, *Fasn*, and *Srebp1*. Additionally, a negative correlation was also observed between CDCA and *Srebp1*.

### 3.4. 1-Kestose Intake Altered the Microbiota in Cecal Contents

**Figure 5:**
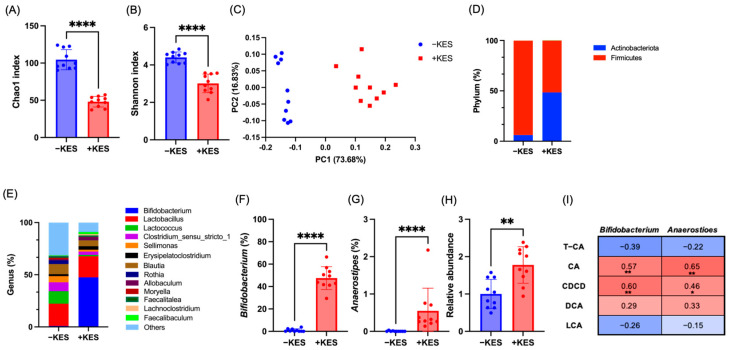
Alpha diversity indices: (**A**) Chao1, (**B**) Shannon, and (**C**) principal component analysis score plot; relative abundances of gut microbiota at the (**D**) phylum and (**E**) genus levels; abundances of (**F**) *Bifidobacterium* and (**G**) *Anaerostipes*; and (**H**) predicted BSH gene abundance in the cecal contents of rats. (**I**) Correlation analysis between gut microbiota and bile acids was performed using Spearman’s correlation. Data are presented as the mean ± standard deviation (n = 10 animals per group). * *p* < 0.05, ** *p* < 0.01, **** *p* < 0.0001. +KES, 4% (*w*/*v*) 1-kestose treatment; −KES, control; BSH, bile salt hydrolase.

We evaluated the gut microbiota using 16S rRNA amplicon sequencing. The alpha diversity indices, Chao1 and Shannon, were significantly reduced in the +KES group (Figure 5A,B). Beta diversity analysis using Weighted UniFrac revealed a significant difference between the two groups (Figure 5C) (*p* = 0.001). At the phylum level, *Firmicutes* were predominant in the -KES group, whereas *Actinobacteria* were dominant in the +KES group (Figure 5D). At the genus level, *Bifidobacterium*, *Allobaculum*, and *Faecalibaculum* populations were significantly increased, whereas *Lactococcus* and *Rothia* populations were significantly decreased in the +KES group (Figure 5E). Furthermore, differential taxon analysis between the two groups using ANCOM identified *Bifidobacterium* and *Anaerostipes* as characteristic genera in the +KES group (Figure 5F,G). Functional gene prediction analysis of the gut microbiota using PICRUSt2 showed that the level of bile salt hydrolase (BSH) was significantly increased in the +KES group (Figure 5H). Spearman’s correlation analysis was used to evaluate the associations between bile acids and the relative abundances of *Bifidobacterium* and *Anaerostipes*, which differed significantly between the two groups. As a result, positive correlations were observed between these bacteria and the bile acids CA and CDCA.

## 4. Discussion

Previous studies have reported that the intake of prebiotics, including 1-kestose, reduces blood cholesterol and triglyceride levels [28,37]. However, the mechanism underlying the blood lipid reduction by 1-kestose remains unclear.

In this study, 1-kestose intake reduced blood cholesterol and triglyceride levels in rats fed a high-fat diet. Additionally, *Gpam* and *Fasn*, which are involved in triglyceride synthesis, were downregulated after 1-kestose intake. Furthermore, the transcription factor *Srebp1* was downregulated [9], suggesting that the reduction in triglyceride levels may be associated with the downregulation of *Srebp1*. An investigation of cholesterol synthesis and metabolism-related genes in the liver, the primary source of cholesterol, revealed that 1-kestose intake significantly downregulated *Cyp7a1*, the rate-limiting enzyme for bile acid synthesis from cholesterol. In this study, 1-kestose intake led to a reduction in body weight and food intake, which may have contributed to the improvement in lipid metabolism. Previous reports have shown that the intake of fructooligosaccharides containing 1-kestose increased GLP-1 production and decreased food intake in rats [38]. Therefore, it is suggested that the reduction in body weight and food intake observed in this study may be partly due to increased GLP-1 expression by 1-kestose intake.

Except for certain ones, bile acids are agonists of FXR and regulate Cyp7a1 expression in the liver via mediators such as a small heterodimer partner [39]. Furthermore, Fxr is pharmacologically activated to reduce plasma cholesterol levels [40]. In the present study, bile acids in the cecal contents were evaluated using LC-MS, and the levels of deconjugated bile acids, such as CA, CDCA, and DCA, were found to be increased with 1-kestose intake. Deconjugated bile acids are less likely to be reabsorbed, leading to a reduction in the bile acid pool, which subsequently enhances *Cyp7a1* expression [41]. In this study, a positive correlation was observed between *Cyp7a1* expression and CA levels. These findings suggest that changes in bile acid metabolism by 1-kestose intake may have influenced hepatic *Cyp7a1* gene expression. In addition, the pH of the cecal contents decreased. Previous studies have reported that 1-kestose intake increases short-chain fatty acid levels, which may contribute to the reduction in pH [24]. A decrease in pH may hinder the formation of bile acid micelles, potentially leading to reduced bile acid reabsorption [42]. Additionally, the suppression of micelle formation may have reduced the absorption of cholesterol and triglycerides in the intestine, resulting in decreased blood lipid levels.

Gut microbiota dominated by *Firmicutes* is a characteristic feature of dysbiosis observed in obese individuals and in rats fed a high-fat diet [43,44]. In our study, 1-kestose intake reduced the abundance of *Firmicutes*, potentially improving dysbiosis caused by HFD. The results of the beta diversity analysis, which assessed the overall differences in the gut microbiota composition, indicated that 1-kestose intake altered the gut microbiota. Similar to previous findings [24,45], the identification of differential taxa using ANCOM revealed a significant increase in the abundance of *Bifidobacterium* and *Anaerostipes*, both of which are short-chain fatty acid-producing bacteria, in the +KES group. These bacteria have previously been reported to metabolize 1-kestose, suggesting that their growth may have been promoted by its utilization [45]. In addition, a positive correlation was observed between *Bifidobacterium* and the bile acids CA and CDCA. Furthermore, *Bifidobacterium* possesses BSH activity [46]. Indeed, the administration of *Bifidobacterium* has been reported to increase CA levels [47]. These findings suggest that 1-kestose promotes the growth of bacteria with BSH activity and potentially alters bile acid metabolism in the gut. Notably, a previous study on the intake of 1-kestose-containing water over a period of 19 weeks showed a slight increase in *Bifidobacterium* abundance [23]. Therefore, the effects of 1-kestose intake on the gut microbiota may differ between short- and long-term consumption.

The limitation of this study is that, while a correlation between changes in the gut microbiota, metabolites, and host metabolic changes is suggested, causality and detailed mechanism remain unclear. It is necessary to use germ-free mice and other models to clarify the causal relationship with the gut environment and to analyze other lipoproteins and gene expressions related to bile acid synthesis and inflammation. Furthermore, it is possible that improvements in lipid metabolism were also influenced by pathways independent of bile acid metabolism, such as those mediated by TGR5 or changes in other gut microbial metabolic functions, which should be clarified in future studies [48]. Additionally, to determine whether the results of this study are specific to 1-kestose, a comparison with other prebiotics is required.

## 5. Conclusions

In high-fat diet-fed mice, the reduction in blood cholesterol and triglyceride levels induced by 1-kestose intake may be attributed to alterations in the gut microbiota, which subsequently affect bile acid metabolism and downregulate hepatic cholesterol and triglyceride synthesis-related genes. This study highlights the potential of 1-kestose, the simplest component of fructooligosaccharides, in reducing cholesterol and triglyceride levels. This effect could contribute to reducing the risk of cardiovascular diseases and their associated complications, positioning it as a highly promising dietary intervention.

## Figures and Tables

**Table 1:** Primer sequences used for quantitative reverse transcription polymerase chain reaction.

Gene		Sequence (5′–3′)
*Abcg5*	F	CGCAGGAACCGCATTGAAA
	R	TGTCGAAGTGGTGGAAGAGCT
*Abcg8*	F	GATGCTGGCTATCATAGGGAGC
	R	TCTCTGCCTGTGATAACGTCGA
*Srebp2*	F	GGTACGCTGGTTACTCAAAAAGG
	R	CCCTCGCACTGCTCTTAGCT
*Cyp7a1*	F	CCAAGTCAAGTGTCCCCCTCTA
	R	GACTCTCAGCCGCCAAGTG
*Baat*	F	CTGTCGAACTACGGTTTTGGCCAA
	R	TCAGGCCTGTGACCCGGATA
*Gpam*	F	CCTGTGGGCATCTCGTATGAT
	R	TTCCGCAGCATTCTGATAAC
*Fasn*	F	CTGCTGCGGGCCAAGACAG
	R	GCTGTGGATGATGTTGATGATAG
*Srebp1*	F	GCAAGGCCATCGACTACATC
	R	TTTCATGCCCTCCATAGACAC
*Gapdh*	F	CTTCACCACCATGGAGAAGGC
	R	GGCATGGACTGTGGTCATGAG

*Abcg5*, ATP-binding cassette subfamily G members 5; *Srebp2*, sterol regulatory element-binding protein-2; *Cyp7a1*, cytochrome P450 family 7 subfamily A member 1; *Baat*, bile acid-CoA: amino acid N-acyltransferase; *Gpam*, glycerol-3-phosphate acyltransferase mitochondrial; *Fasn*, fatty acid synthase; *Gapdh*, glyceraldehyde-3-phosphate dehydrogenase.

## Data Availability

The raw sequencing data generated for this study have been deposited in the National Library of Medicine under the accession number PRJNA1234880. The dataset is publicly available at https://www.ncbi.nlm.nih.gov/bioproject/1234880 accessed on 13 March 2025. Further details can be obtained from the corresponding author upon reasonable request.
